# Supplementary material for: The National Implementation of a Community Pharmacy Antimicrobial Stewardship Intervention (PAMSI) through the English Pharmacy Quality Scheme 2020 to 2022
Source: Antibiotics (Basel). 2023 Apr 21;12(4):793. doi: 10.3390/antibiotics12040793 (PMC10135021; doi:10.3390/antibiotics12040793)
Supplement: Supplementary file 1 [file antibiotics-12-00793-s001.zip › antibiotics-2249008-supplementary.pdf]

Supplementary file S1: TARGET Antibiotic Checklist data entry

This form is for you to input the data collected on the TARGET Antibiotic Checklist for community pharmacies as part of the Pharmacy Quality Scheme 2021-22.

Each page of this form will correspond with each of the 4 pages of the Antibiotic Checklist for individual patient entries. Please double check each answer before proceeding to ensure your form is as accurate as possible. If submitting more than one form, please direct back to the original link after you have submitted your first one.

By completing this form, you understand and agree that PHE/UKHSA may use and share information you provide in relation to this claim internally and with Department of Health and Social Care (DHSC), NHS England and NHS Improvement (NHSEI), and NHS Counter Fraud Authority (NHSCFA) for the purposes of the prevention, detection, loss measurement, investigation and prosecution of fraud or any other unlawful activity affecting the NHS and for the purposes of contract management, provider assurance and service development. The data may be used to make decisions about antimicrobial stewardship to help prevent the development of current and future antimicrobial resistance.

If you have any questions about this form, please contact ESPAUR@phe.gov.uk

Q1 What is your pharmacy ODS (F) code?

Q2 What is your pharmacy's full postcode?

Q3 Please provide your pharmacy shared NHS email address (e.g., Xpharmacy@nhs.net) This is necessary for confirmation.

Q4 If you would like confirmation to go to an additional email address, please share **one** below (optional).

Q5 What date did you start this project in your pharmacy? (dd/mm/yyyy)

Antibiotic Checklist pages 1–2: For patients

Q6 Date Antibiotic Checklist was completed with patient (dd/mm/yyyy)

Q7 Complete the following information about the patient:

	Yes	No	Don't know/missing
Were the antibiotics for the patient?			
Was the patient taking any other medicines?			
Had the patient taken the same antibiotics in the last 3 months?			
Was the patient allergic to any antibiotics?			

Q8 Which of the following common infections did the patient have?

Chest Throat Ear Urine Tooth Skin Don't know/ missing Other

If other please state

Q9 Did the patient have any of these risk factors?

Problem with kidney function | Problem with liver function | Breast feeding | Pregnant | Over 65 | None

Q10 Had the patient had the flu vaccine this winter season?

--Click Here--

Yes

No

Don't know

Not applicable

Q11 If the patient requested a language other than English, please indicate here. Do not include any patient identifiable data.

Q12 This question requires two answers per row. For each of the following statements, tick how the patient answered (if they understood the statement or not) and if the pharmacy team provided information to the patient on this topic.

	Patient ticked 'yes they understood'	Ticked 'no they did not understand'	Missing data for patient	Pharmacy team provided advice	Pharmacy team did not provide advice	Missing data for pharmacy team
I know what to do if I miss a dose of my antibiotics						
I know whether my antibiotics should be taken with or without food						
I know why I must take my antibiotics as advised by my doctor, nurse of pharmacist						
I know about side effects that I might get from my antibiotics						
I know whether I need to avoid alcohol whilst I am taking my antibiotics						
I know why I must never share my antibiotics or keep for later use						
I know how long my symptoms are likely to last						
I know when I should seek further help with my infection						
I know why I must return any unused antibiotics to the pharmacy						

Antibiotic Checklist pages 3–4: For pharmacists

Q14 The pharmacy team checked:

	Yes	No	N/a	Don't know/missing
for patient allergies, risk factors, other medication interactions				
treatment with the same antibiotic in the previous 3 months				
that the antibiotic was in line with the local guidance				
that the antibiotic was appropriate for the infection indicated				
that the dose was correct for the indication and patient				
that the duration was correct for the indication				

Q15 Was the prescriber contacted about this prescription?

--Click Here--

- Yes
- No
- Not applicable
- Don't know/missing

If yes, what was the reason for contacting and outcome of contacting? Do not include any patient identifiable data.

Q16 The following antibiotics were dispensed

Q17 The pharmacy team:

	Yes	No	Don't know/ Missing	Not applicable
discussed antibiotic resistance with the patient/carer as the patient had taken the same antibiotic in the last 3 months				
checked the patient responses to the statements overleaf and gave advice as required				

Q18 Was the patient given an infection self-care leaflet?

Yes No

Not applicable

Don't know/Missing

Q19 If yes, which of the following patient information leaflets were given to the patient (if known)?

Q20 When administering the flu vaccine (if applicable), the pharmacy team:

	Yes	No	N/a	Don't know/missing
discussed flu vaccine eligibility with the patient				
gave the patient the flu vaccine on site				

Q21 The Antibiotic Checklist has not been fully completed for this patient because:

--Click Here--

- the patient's representative did not know the information
- the antibiotics are supplied by delivery service
- the antibiotics are already dispensed
- the patient declined
- Other

Q22 Any other information or text on the checklist to disclose. Do not include any patient identifiable data.

Q23 Can we contact you about other Antimicrobial Stewardship projects?

Yes No N/a

Supplementary file S2: follow up questionnaire for 2021/22 PQS

Pharmacy staff feedback on AMS activities

Dear Colleague,

We invite you to complete this short survey to provide feedback about your Antimicrobial Stewardship (AMS) activities and using the Antibiotic Checklist in your community pharmacy as part of the 2021/22 Pharmacy Quality Scheme. This will also support the evaluation of the criteria and help us understand how we can further support community pharmacy teams. We welcome responses from all community pharmacy teams, including those that did not participate in the PQS as we would like to understand the barriers.

We do not collect identifiable data, and responses are confidential, not attributable and are treated in compliance with GDPR. The findings will be used to inform reports, peer-review publications and shared learning; however, no individual responses will be identifiable.

Q1 Do you understand the purpose of this survey and consent to continue?

Yes

No - please review the information above and contact ESPAUR@phe.gov.uk if you have questions

Q2 Did you as an individual contribute to using the TARGET Antibiotic Checklist as part of the Antimicrobial Stewardship criteria of the 2021/22 Pharmacy Quality Scheme?

Yes

No

About you and your AMS actions

Q3 What is your role? (Please tick most appropriate box)

--Click Here--

- Pharmacist
- Pharmacist manager
- Trainee pharmacist
- Pharmacy owner
- Locum pharmacist
- Member of healthcare counter team (e.g., healthcare assistant or healthcare partner)
- Member of dispensary team/dispenser

If other, please specify

Q4 Which type of pharmacy do you work in?

--Click Here--

- Independent
- Large multiple
- Small multiple
- Health Centre

If other, please specify

Q5 What is the location of your pharmacy?

--Click Here--

- Local community
- High street
- Co-located with GP practice/healthcentre
- Retail park
- Hospital

Q6 Did your pharmacy have a local Antimicrobial Stewardship (AMS) Action plan prior to you using the Antibiotic Checklist as part of the 2021/22 Pharmacy Quality Scheme?

--Click Here--

- Yes
- No
- Not applicable
- Don't know

Q7 Has your pharmacy created an AMS action plan since using the Antibiotic Checklist?

--Click Here--

- Yes
- No and don't plan to
- No but plan to
- Not applicable

Q8 Has your pharmacy updated your existing AMS action plan to include the Antibiotic Checklist?

--Click Here--

- Yes
- No and don't plan to
- No but plan to
- Not applicable

Q9 Do you have access to a hard copy of the Antibiotic Checklist (sent to all English Community Pharmacies in March 2021)

--Click Here--

- Yes, we have been using the laminated copies sent in March 2021
- Yes, we printed our own copies
- No
- Not applicable

Q10 Do you have access to local antibiotic guidance to support you in checking the appropriateness of prescriptions?

--Click Here--

- Yes
- No
- No but use other guidance
- Not applicable
- [illegible]

Q11 Do you have access to infection self-care leaflets to share with patients (e.g., TARGET leaflets)? See examples here.

--Click Here--

- Yes
- No
- Don't know

Q12 If yes, how do you provide these to patients?

--Click Here--

- Printed copy - printed in house
- Printed copy - provided by an organisation
- Send website URL direct to patient (texted or emailed)
- Directed to website URL (e.g. on a poster, or verbal)

Q13 How did you use the Antibiotic Checklist with patients?

- Patient completed the questions in the 'for patients' section on a hard copy of the Antibiotic Checklist
- Pharmacy staff asked the questions verbally and inputted answers onto a hard copy of the Antibiotic Checklist
- Pharmacy staff asked the questions verbally and inputted answers directly onto the online form

If another way, please state

Q14 What patients did you decide to use the Antibiotic Checklist with?

- We tried to use it with all patients with an antibiotic prescription
- We targeted specific patient groups
- When we were less busy
- Other

Other please state

What types of patients?

About the Antibiotic Checklist and submission of data

Q15 Did you have any issues contacting a prescriber about the appropriateness or safety of an antibiotic prompted by using the Antibiotic Checklist?

Yes

No

Didn't need to contact the prescriber during the PQS period

Q16 Please describe the issues faced while contacting a prescriber.

Q17 How, if at all, have you continued to use the Antibiotic Checklist and TARGET patient information leaflets after completing the PQS?

	Antibiotic Checklist	TARGET patient information leaflets
We have continued to use it in our daily practice		
We sometimes use it, but it is not part of daily practice		
We do not use it but we have continued to use the principles (e.g., tailoring advice to patients)		
We have not used it since completion of the PQS		
Other		

If other, please state

Q18 What are the barriers to continued use the Antibiotic Checklist after completing the PQS? If no barriers please state.

Q19 I/our team feel that using the Antibiotic Checklist as part of the AMS criteria of the PQS…

	Strongly agree	Agree	Neutral	Disagree	Strongly disagree	N/a
was valuable for patient care						
improved our confidence to query antibiotic prescriptions with prescribers						
led to us contacting prescribers more often to discuss the appropriateness of antibiotics						
led to us embedding/planning to embed the Antibiotic Checklist into everyday practice						
was helpful for identifying interventions						

Q20 The time it took to complete the Antibiotic Checklist and provide tailored adherence and self -care advice to each patient is...

	Strongly Agree	Agree	Neutral	Disagree	Strongly Disagree	N/A
feasible in every day practice						
justified by the benefits to keeping antibiotics working						

Q21 The Antibiotic Checklist submission process for the PQS through the online portal…

	Strongly Agree	Agree	Neutral	Disagree	Strongly Disagree	N/A
was clear and straightforward to complete						
was feasible in terms of time required						

Q22 When did your staff usually input data from Antibiotic Checklists into the online portal?

--Click Here--

Submitted the day’s data at the end of each shift

After every relevant consultation

All at once at the end of the 4- or 8-week collection period

Ad hoc

Q23 Please provide any other reflections on the Antibiotic Checklist submission process and data entry for the PQS. Please consider sharing what went well and what didn't go so well.

Q24 Please add any further reflections on your AMS action plan.

Q25 Would you be happy to be contacted about Antimicrobial Stewardship in the future? (You can opt out at any time.)

Yes

No

Please provide email address

Demographic questions

Please submit your answers before leaving this page

Q28 Region of England:

--Click Here--

- East of England
- London
- East Midlands
- North East
- North West
- South East
- South West
- Yorkshire and The Humber
- Don't know
- [illegible]

Q29 Please provide the first half (3–4 digits) of your pharmacy postcode. This is to allow us to ensure good representation across the country.

Q30 Number of years of pharmacy experience:

Q26 Your age:

Under 25	25-24	35-44	45-54	55+	Prefer not to say	N/A

Q27 Gender:

Male	Female	Other	Prefer not to say	N/A

Supplementary Figure S1: Flowchart showing completion of the Antibiotic Checklist, numbers of submissions that were incomplete, and the reasons why

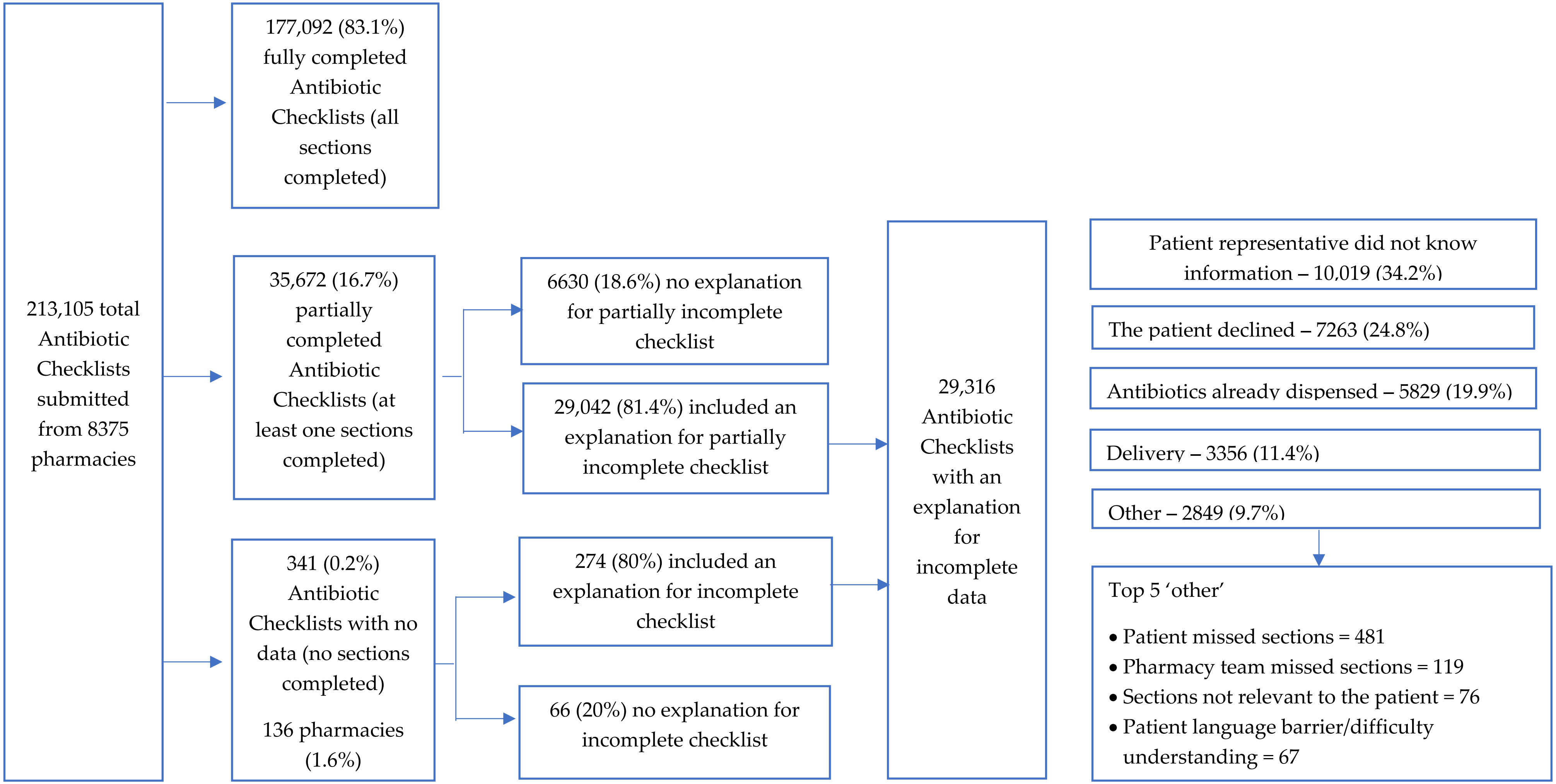

Supplementary Table S1: Demographics of pharmacy staff respondents of the follow-up questionnaire

Variable	Number	%
**Role**		
Pharmacist manager	42	40%
Pharmacist	39	37%
Pharmacy technician	9	9%
Pharmacy owner	6	6%
Member of dispensary team/dispenser	5	5%
Member of healthcare counter team	1	1%
Head office for a multiple	1	1%
Non-pharmacist manager	1	1%
Trainee pharmacist	1	1%
**Age**		
Under 25	3	3%
25-34	16	16%
35-44	29	28%
45-54	28	26.5%
55+	24	23.5%
Undisclosed	4	4%
**Gender**		
Female	67	66%
Male	33	31%
Undisclosed	3	3%
**Pharmacy type**		
Large multiple	40	39%
Independent	36	35%
Small multiple	26	23%
Health centre	3	3%
**Pharmacy location**		
Local community	65	62%
High street	23	23%
Co-located with GP or health centre	13	12%
Retail park	2	2%
Other	1	1%
**Region**		
North West	17	16%
East of England	15	14%
South East	12	12%
South West	12	12%
West Midlands	14	13%
London	11	11%
East Midlands	9	9%
North East	8	8%

Yorkshire and The Humber	6	6%
Undisclosed	1	1%

Supplementary Table S2: Thematic analysis of open-ended responses about the value of the PQS and factors affecting continued use of the resources

Theme	Subtheme	Count	Example quote
Pharmacy resources	Time	43	*Staff handing our rxs don't have the time. We can motivate them to do it for a limited period of time but to do this daily with every patient would be very time-consuming and not practical.*<br><br>*It's time consuming and potentially counterproductive - it could easily undermine the relationship between GP and patient (at a time when public opinion of GPs is already low), and also between pharmacies and GP surgeries. Also, have you TRIED to get hold of a GP in a timely fashion? You generally will be called back eventually, but it's usually hours rather than minutes, which means… your patient sits there waiting? You tell them they can't have their antibiotics, and they must come back at an indeterminate point in the future?*<br><br>*main barriers are time needed to consult with patients who are always in a hurry. staff training introduced but still takes a lot of time.*<br><br>*We just have the time and resources to spend 5 minutes with a patient*
	Short-staffed	12	*Staffing problems mean that dispensers have very little time to interact with patients, counter staff are newly recruited and are still learning the basics.*<br><br>*short staffing, too busy, so sometimes have to save time for other services*<br><br>*Time. We are a busy pharmacy and staff are quite stretched. This means the counter staff who are not all clinically trained to understand and effectively complete the checklist are often handing out prescriptions. Patient's are not always willing and do not always have time to complete the checklist.*

	Busy pharmacy with little time for additional services	9	*It is really valuable and important but like every part of community pharmacy, every resource is stretched. We are constantly just trying to keep the essentials done*<br><br>*time and space available in the pharmacy- not easy to talk with screens etc and patients do not wish to enter the consultation room all the time. a simplified approach of 3-4 questions/ advice points is preferred but same principles*
	Printed resources	5	*The AMS action plan involves handing out leaflets to patients and being sent a supply of these periodically will certainly encourage further usage or continued use.*
Patient factors	Patients not willing to wait or too sick to wait	11	*it was bit time consuming, not all patients want to wait so long*<br><br>*Patients who were unwell also didn't want to wait to answer the questions.*
	Lack of patient engagement	7	*The checklist was good when time allowed and when patient's were able to properly go through and listen carefully to key messages. Unfortunately, in some instances patient's were not always engaged and as we were busy we did not have time to provide effective consultations.*<br><br>*Some patients need assistance in completing the form. Some patients do not understand the aim of completing the form, so they refused.*
	Patient not present to ask questions	6	*getting in contact with patients as we do a lot of deliveries and do not have time to continually phone them*<br><br>*Antibiotic prescriptions are picked up by representatives*
	Patient expectation	3	*Although all patients agree that antibiotics should be used appropriately - each individual is not keen to wait and see and prefers to use antibiotics in case they get worse and have to take time out of life to get well*
Believe PQS was valuable and useful	Staff development	7	*Pharmacy staff organising action plans to deliver the service Pharmacy staff refreshed and trained on how to deliver this service and on Antibiotic Awareness for each condition*<br><br>*Great for HCA development and also some customers felt the value of being able to speak to someone in private.*<br><br>*Pharmacy staff organising action plans to deliver the service Pharmacy staff refreshed and trained on how to deliver this service and on Antibiotic Awareness for each condition*

	Patient benefits	5	*I think it would be a good idea for it to become an obligatory practice for each Rx. I think it would help bring the number of prescription for antibiotics down.*<br><br>*Patients more aware about the proper use of antibiotics*<br><br>*patients were happy to speak about their knowledge of antibiotics and only to use when absolutely necessary*
Believe PQS was not valuable or useful	Felt like a 'tick box' activity with no impact on real practice	9	*We always counsel on most points covered anyway so patients saw it as a tick box exercise rather than to benefit them.*<br><br>*It felt like a tick box exercise. Patients who were unwell also didn't want to wait to answer the questions.*<br><br>*The aim is laudable, but this was a PQS hoop-jumping exercise. You're targeting the wrong people, pharmacies are happy to help patients get best use out of the antibiotics they have been prescribed, but the real levers for change are in the hands of the GPs. We don't have the time, staff or authority to makes a significant impact.*
	The AMS role should be performed by GPs, prescribers should be checking appropriateness	5	*Ideally the antibiotic checklist should be carried out by the GP to determine the appropriateness of the antibiotic prescribed.*<br><br>*It can be frustrating that the responsibility for antimicrobial stewardship is being put on the pharmacies as opposed to targeting the actual prescribers who have more the power to reduce inappropriate prescribing however pharmacies are best placed to advise on compliance*
Design of the Antibiotic Checklist and improvements	Design of checklist – too many questions, some not relevant, could be made simpler	9	*There were way too many questions and some of them non sensical to the patient. They could not possibly answer some of the questions prior to having the item dispensed and time to read the leaflet.*<br><br>*It's actually not straight forward to use, particularly as the prescription is usually dispensed and waiting prior to seeing the patient. The form works best if the patient brings in the prescription so the questions can be asked before dispensing the prescription.*
	Difficult to follow process when antibiotics are already dispensed	3	*Although the we understood the principle we found the whole process difficult to complete. We do not have access to information e.g. why the antibiotics were given and sometimes the patients are unaware as well. It seem a bit pointless to talk about antibiotic resistance and that*

	before patient attends pharmacy		*antibiotics aren't always the answer once antibiotics have already been prescribed.*
Embedding principles of PQS	Day to day practice already included principles of checklist	4	*no barriers but didn't find any issues with the prescribing of antibiotics as a result of the checklist and we always counsel on most of the points covered in the checklist anyway so no point wasting paper*<br><br>*The principles are part of our day to day practice and standard procedures, in line with best practice standards, we ensure that all colleagues are aware of the local and stewardship guidelines and incorporate them into our consultations. E.g. medication handout*
	Will continue principles of checklist after PQS	5	*continue to check allergies, advice on food/alcohol, checking indication and quantity matches short courses*<br><br>*It feels more natural and easier to embed into daily practice offering AMS advice to all rather than targeting specific groups.*
